# Sleep Profiles in Eating Disorders: A Scientometric Study on 50 Years of Clinical Research

**DOI:** 10.3390/healthcare11142090

**Published:** 2023-07-21

**Authors:** Alessandro Carollo, Pengyue Zhang, Peiying Yin, Aisha Jawed, Dagmara Dimitriou, Gianluca Esposito, Stephen Mangar

**Affiliations:** 1 Department of Psychology and Cognitive Science, University of Trento, 38068 Rovereto, Italy; alessandro.carollo@unitn.it (A.C.); gianluca.esposito@unitn.it (G.E.); 2 Sleep Education and Research Laboratory, UCL Institute of Education, London WC1H 0AA, UK; pengyue.zhang.22@ucl.ac.uk (P.Z.); peiying.yin.22@ucl.ac.uk (P.Y.); aisha.jawed.15@ucl.ac.uk (A.J.); 3 Department of Clinical Oncology, Imperial College Healthcare NHS Trust, Charing Cross Hospital, London W6 8RF, UK; s.mangar@imperial.ac.uk

**Keywords:** eating disorders, sleep, CiteSpace, scientometrics, systematic review

## Abstract

Sleep and diet are essential for maintaining physical and mental health. These two factors are closely intertwined and affect each other in both timing and quality. Eating disorders, including anorexia nervosa and bulimia nervosa, are often accompanied by different sleep problems. In modern society, an increasing number of studies are being conducted on the relationship between eating disorders and sleep. To gain a more comprehensive understanding of this field and highlight influential papers as well as the main research domains in this area, a scientometric approach was used to review 727 publications from 1971 to 2023. All documents were retrieved from Scopus through the following string “TITLE-ABS ((“sleep” OR “insomnia”) AND (“anorexia nervosa” OR “bulimia nervosa” OR “binge eating” OR “eating disorder*”) AND NOT “obes*”) AND (LIMIT-TO (LANGUAGE, “English”))”. A document co-citation analysis was applied to map the relationship between relevant articles and their cited references as well as the gaps in the literature. Nine publications on sleep and eating disorders were frequently cited, with an article by Vetrugno and colleagues on nocturnal eating being the most impactful in the network. The results also indicated a total of seven major thematic research clusters. The qualitative inspection of clusters strongly highlights the reciprocal influence of disordered eating and sleeping patterns. Researchers have modelled this reciprocal influence by taking into account the role played by pharmacological (e.g., zolpidem, topiramate), hormonal (e.g., ghrelin), and psychological (e.g., anxiety, depression) factors, pharmacological triggers, and treatments for eating disorders and sleep problems. The use of scientometric perspectives provides valuable insights into the field related to sleep and eating disorders, which can guide future research directions and foster a more comprehensive understanding of this important area.

## 1. Introduction

Both anorexia nervosa and bulimia nervosa are classified as eating disorders in subsequent revisions of the third version of the Diagnostic and Statistical Manual of Mental Disorders (DSM) [1]. The main symptom of anorexia nervosa is an excessive fear of weight gain despite being underweight and a distorted experience of body shape. Bulimia nervosa is recurrent binge eating accompanied by a sense of lack of control, but a fear of weight gain that leads to repeated inappropriate compensatory behaviours, such as vomiting [1]. In addition to these two main types of eating disorders, three other conditions are characterised by disordered eating habits, including binge eating disorder, night eating syndrome, and sleep-related eating disorder [2]. The prevalence of eating disorders has been increasing in recent years. The mean prevalence of eating disorders has increased from 3.5% during 2000–2006 to 7.8% during 2013–2018 worldwide [3]. In a study by van Eeden [4], they found that the lifetime prevalence of anorexia nervosa may be as high as 4% in women and 0.3% in men. Although the prevalence of anorexia nervosa is lower in men compared to women, it is also increasing in men. This may be due to the pressure on men by media such as popular magazines in recent years, which has increased the prevalence of anorexia nervosa by making men dissatisfied with their bodies, seeking to achieve a muscular or lean body type [5].

Eating and sleeping are two critical parts of human survival, and these two parts interact with circadian rhythms and hormonal regulation [6]. Sleep problems are characterised by various difficulties, including difficulty initiating sleep, difficulty maintaining sleep, non-restorative sleep Knudsen et al. [7], and related symptoms or disorders such as sleep-disordered breathing, irregular sleep patterns (e.g., circadian rhythm sleep–wake disorders), parasomnia (e.g., sleepwalking, confusional arousals, night terrors, sleep talking, and nightmares), and sleep-related movement disorders (e.g., restless leg syndrome). These issues with sleep can be assessed using various methods, including polysomnography, actigraphy, sleep diaries, Multiple Sleep Latency Testing, the Epworth Sleepiness Scale, the Fatigue Severity Scale, and the Insomnia Severity Index [8]. Sleep deprivation has been shown to have a negative impact on hormones that control appetite, namely leptin and ghrelin, which can lead to an abnormal increase in appetite [6]. In sleep studies conducted with patients diagnosed with anorexia and bulimia, it has been shown that people have reduced sleep maintenance and increased night-time awakenings [1]. In addition, other related studies have found that patients with anorexia nervosa experience sleep problems more often than those with bulimia nervosa, including symptoms of insomnia [2]. The alleviation of sleep disturbances associated with different eating disorders has been reported in several studies, although the number of such studies is still relatively small [9,10]. Sleep management and assessment in this clinical population are yet to be established as there appears to be a lack of randomised control clinical studies and patient pathways for sleep management.

### The Current Study

Recent studies are exploring the relationship between eating and sleep disorders, as well as strategies to alleviate them. However, this field still needs to be systematically reviewed. Some literature reviews of eating disorders are incomplete or limited in scope (i.e., only individual eating disorder syndromes are discussed, e.g., [6]). Furthermore, there are conflicting findings across different studies (e.g., [1,2]). Therefore, a different research approach will be needed to incorporate the full range of studies in the field systematically.

This article will use the scientometric approach to reviews. Scientometrics lies in the intersection between scientific mapping (i.e., the visual inspection of the temporal evolution of a research domain) and bibliometric analysis [11,12]. The scientometric approach allows for systematically reviewing the existing literature in a data-driven way and uses quantitative relationships between publications to map relevant areas of knowledge [13,14]. This study aims to adopt the scientometric approach to review the literature on eating disorders and sleep. The current work aims to identify impactful documents as well as major thematic domains in the literature by means of a document co-citation analysis (DCA) (as in [15,16]). In doing so, research gaps and possible future lines of research will be discussed.

## 2. Materials and Methods

### 2.1. Data Collection from Scopus

The current review was not previously registered and no protocol was previously prepared. The current manuscript mirrors the same analytical approach as previously published scientometric reviews [17,18,19].

According to the standard and established scientometric procedures [20], data collection for the current literature was performed on Scopus on 26 January 2023. Scopus was chosen as the main source because, as compared to similar engines, it provides wider coverage in terms of the number of indexed journals [21]. The search string “TITLE-ABS ((“sleep” OR “insomnia”) AND (“anorexia nervosa” OR “bulimia nervosa” OR “binge eating” OR “eating disorder*”) AND NOT “obes*”) AND (LIMIT-TO (LANGUAGE, “English”))” was applied in order to collect a comprehensive and accurate collection of studies on sleep in eating disorders. Considering the rigour of the analysis, only publications written in English were included in this search to ensure that the collection was of international scientific standard [22]. Through this approach, 727 publications from 1971 to 2023 were found and downloaded in total. The sample of documents downloaded from Scopus was analysed through the *bibliometrix* package for R [23]. The *bibliometrix* package for R allows the identification of the main information in the sample of documents, the citing documents with the highest number of citations, the most productive authors, the countries that appear the most in authors’ affiliation as well as cross-country collaborations, main sources, and main keywords in the field. A keyword co-occurrence analysis was computed among the top 100 most occurring keywords. Based on the keyword co-occurrence frequency, the *bibliometrix* package automatically detects the main groups of keywords that appear together.

### 2.2. Data Import on CiteSpace

Downloaded publications were imported into the software CiteSpace (version 6.1.R6 64-bit Premium) [24]. CiteSpace was chosen over other softwares because it allows us to conduct a DCA to identify the main thematic clusters of research within a domain of science. When importing the data, CiteSpace identified 42,503 references cited by the 727 documents downloaded from Scopus, of which 41,798 (98.34%) were considered valid in terms of format. Although some references were missed, the data loss rate of 1.66% was within the acceptable range of 1–5% and is therefore negligible [25]. To avoid duplicate or identical articles and references, the ’Remove Alias’ function was turned on.

### 2.3. Document Co-Citation Analysis (DCA)

In order to identify the main research domains in the literature on sleep in eating disorders, DCA was applied to the downloaded papers and their valid references. DCA is a bibliometric analysis method and can be used to visualise and assess scholarly outputs across diverse disciplines. DCA is based the frequency with which two or more papers are co-cited in the same publication [26]. High co-citation frequencies can reveal a shared thematic domain, and such co-citation patterns can detail the relationships between key concepts, methods, or experiments in the field [27]. CiteSpace DCA constructs a network by including the individual papers as single nodes and their co-citations as edges. The strength of co-citation is then used as the weight for the edges.

**Figure 1:**
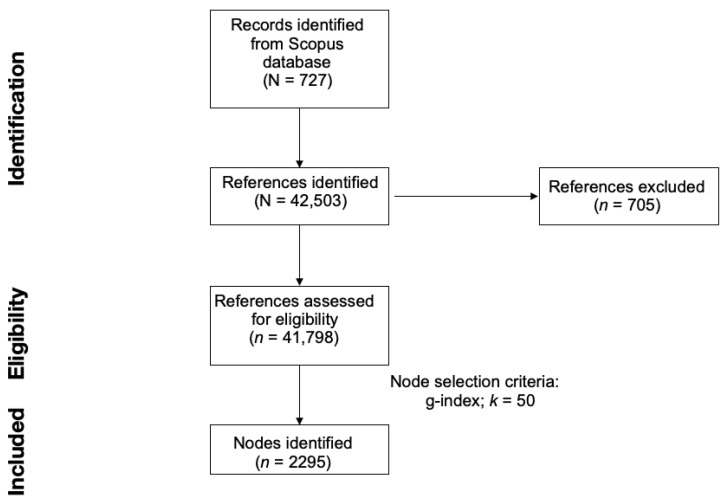
Preferred reporting items for systematic reviews (PRISMA) flowchart for literature search and reference eligibility.

In order to achieve a well-balanced data network, DCA parameter optimisation was conducted considering three node selection criteria: g-index, TOP N and TOP N%. The g-index is used to measure an author’s citation score. Since the g-index takes into account both less cited papers and particularly highly cited papers, it can reduce more biases than the h-index and is therefore adopted as an improvement of the h-index [28]. Top N and Top N%, the other two criteria, identify the N or N% most referenced documents within a specified time interval (i.e., time slice) [20]. In this study, the time slice was set at one year per slice, as in many other scientometric studies [29,30]. In order to establish an optimal DCA network, the node selection criteria were tested and controlled as follows: g-index with k = 25, 50, 75, 100, Top N with N = 50, Top N% with N = 10. The node selection criterion and the value for the scaling factor were selected from the main page of the CiteSpace software. After comparing the generated networks by taking into account the final number of nodes, number of links, number of clusters, modularity, and silhouette, a g-index with k = 50 was identified as the ideal parameter for performing the DCA and generating the final network. The literature search and the generation of the DCA network are summarised in Figure 1.

### 2.4. DCA Network Evaluation Metrics

Metrics used to evaluate the CiteSpace DCA network can be divided into structural and temporal metrics.

Structural metrics include modularity-Q, silhouette scores, and betweenness centrality. Modularity-Q reflects the degree to which the network can be broken down into single modules or clusters and is a measure that evaluates and compares the quality of different clusterisations [31,32]. It has a range from 0 to 1. The higher the modularity-Q value, the greater the divisibility and the better structure of a network [33]. Silhouette score (ranging from −1 to 1) represents a cluster’s inner consistency and the level of separation from other clusters, with a higher value showing a higher degree of isolation and inner consistency of the cluster [34,35]. Betweenness centrality, ranging between 0 and 1, indicates the extent to which a single node connects two other random nodes in the network [20,36]. A high betweenness centrality means the work is profound and pathbreaking [34].

Temporal metrics consist of citation burstness and sigma. Citation burstness (ranging from 0 to infinity) is gained from the calculation with Kleinberg’s algorithm and indicates abrupt spikes in publications’ number of citations over time [37]. It can be used to uncover publications that have received significant attention and general recognition [38]. Sigma is calculated with betweenness centrality and citation burstness using the equation ${\left(centrality+1\right)}^{burstness}$. A higher value of sigma means that a piece of literature has a higher impact on the whole network and is more innovative and significant [39].

## 3. Results

### 3.1. Bibliometric Analysis on the Citing Documents

From the bibliometric analysis of the citing documents downloaded from the Scopus platform, it emerged that the literature on sleep and eating disorders grew from 1971 to 2023 with an annual growth rate of 2.87%. On average, documents received a number of 31.61 citations. In the sample of citing documents, the most cited were Hofmann et al. [40] (total citations = 1485; total citations by year = 123.8) and Gustavsson et al. [41] (total citations = 1151; total citations by year = 88.5).

In the sample, a total of 3112 authors were identified, of whom Schenck CH (*n* = 24 documents), Latzer Y (*n* = 13 documents), and Howell MJ (*n* = 12 documents) were the most productive.

Based on the corresponding authors’ affiliations, the countries most involved in the study of eating disorders and sleep were found to be the United States of America (*n* = 188 documents; Single Country Publications (SCP) = 165; Multiple Country Publications (MCP) = 23), Italy (*n* = 56 documents; SCP = 39; MCP = 17), and France (*n* = 31 documents; SCP = 27; MCP = 4).

The main sources for articles on sleep and eating disorders were the *International Journal of Eating Disorders* (*n* = 17 documents), the *Eating and Weight Disorders* (*n* = 16 documents), and *Sleep Medicine* (*n* = 16 documents).

**Figure 2:**
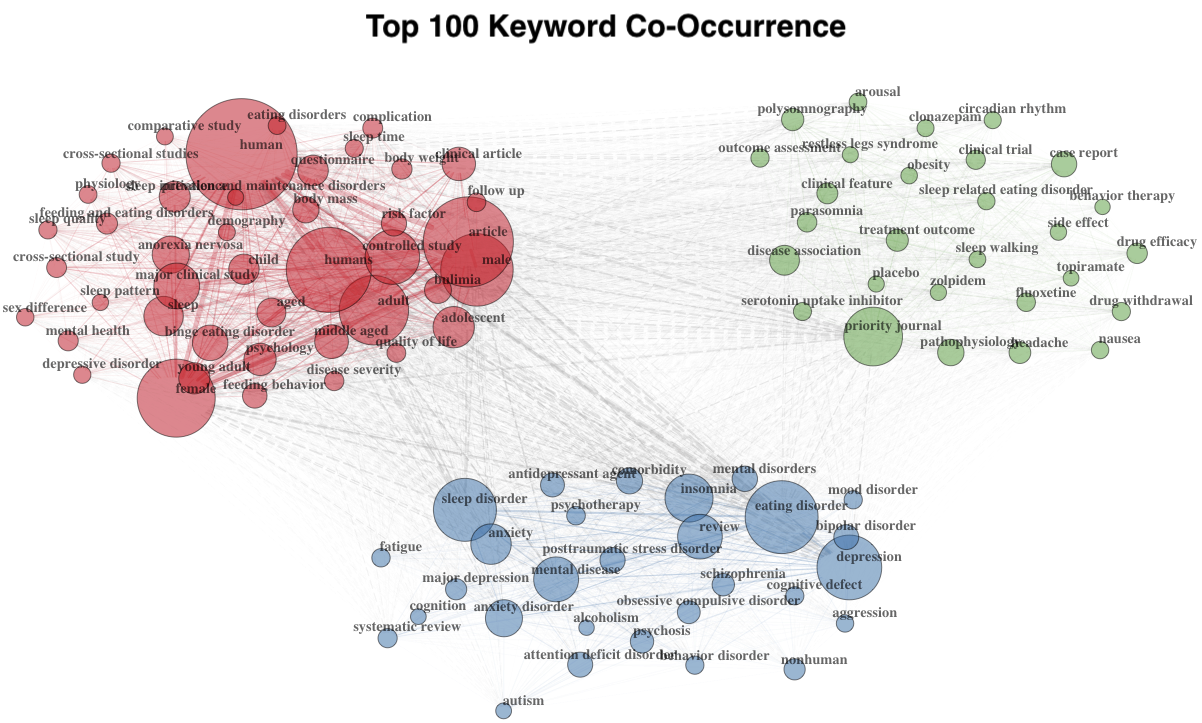
Co-occurrence analysis of the 100 most frequent keywords in the literature on sleep and eating disorders. The figure was generated with the *bibliometrix* package for R [23]. Three clusters of keywords were automatically identified by the package based on the keyword co-occurrence frequencies.

Finally, in terms of document content, 1485 keywords were identified. In particular, the ten most frequent keywords in the manuscript were *eating disorders* (*n* = 70 documents), *depression* (*n* = 64 documents), *sleep* (*n* = 51 documents), *anxiety* (*n* = 34 documents), *anorexia nervosa* (*n* = 33 documents), *sleep-related eating disorder* (*n* = 31 documents), *insomnia* (*n* = 30 documents), *eating disorder* (*n* = 27 documents), *night eating disorder* (*n* = 27 documents), and *mental health* (*n* = 23 documents). The list of the 100 most frequent keywords in the citing documents is provided in the Supplementary Materials of the manuscript. Patterns of co-occurrence among the 100 most used keywords are displayed in Figure 2.

### 3.2. Document Co-Citation Analysis

**Figure 3:**
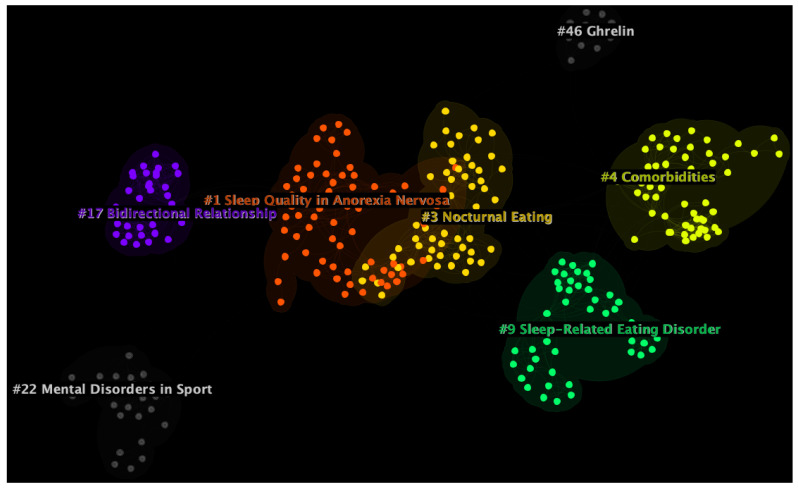
Document co-citation analysis network of all studies on sleep in eating disorders. Seven major clusters were identified. The image was generated with CiteSpace software [24].

The optimal DCA network included 2295 nodes and 6910 links (average = 3.01 connections per node; see Figure 3). The Modularity Q of the network was 0.974, illustrating that the network was highly divisible into clusters, while the mean cluster silhouette was 0.9772, which showed that these clusters were highly internally consistent.

Seven major thematic clusters were identified in the network. The three largest clusters were cluster #1 (size = 69; silhouette = 0.956; mean publication year = 2014), cluster #3 (size = 57; silhouette = 0.970, mean publication year = 2007), and cluster #4 (size = 56, silhouette = 0.973, mean publication year = 2010). Based on their silhouette scores, the most homogenous clusters were cluster #17 (size = 34; silhouette = 1.000; mean publication year = 2015), cluster #46 (size = 11; silhouette = 0.999; mean publication year = 2010), and cluster #22 (size = 25, silhouette = 0.995, mean publication year = 2013). Moreover, cluster #17 was also the most recent cluster in the network, followed by cluster #1 and cluster #22. Finally, the three earliest clusters were cluster #9 (size = 48; silhouette = 0.991; mean publication year = 2005), cluster #3, and cluster #46. All clusters were initially automatically labelled using CiteSpace’s log-likelihood ratio (LLR) algorithm. LLR was chosen because it provides the most accurate labels compared to other automated methods. However, LLR in some cases might lack accuracy as compared to manual labelling [16]. Therefore, a qualitative inspection of clusters was conducted by the authors and an alternative label was suggested when appropriate [42]. More detailed information on all seven major clusters can be found in Table 1.

### 3.3. Impactful Documents

Furthermore, a total of nine documents with significant citation bursts were detected. These nine documents include five research articles, two review studies, one diagnostic manual, and one editorial. The article with the strongest citation burstness was authored by [43] with a citation burst of 8.3575 from 2007 to 2012 (duration = 5 years). This article suggested that sleep-related eating disorders and nocturnal eating syndrome share some similarities, including the fact that both involve nocturnal eating. However, they are distinctly different in terms of the underlying sleep disorder and associated psychological symptoms. The following documents in terms of citation burstness were authored by Morgenthaler and Silber [44] (burst strength = 6.7376; duration = 4 years) and by Howell et al. [45] (burst strength = 5.9064; duration = 2 years). The metrics for all nine documents with a citation burst can be found in Table 2.

## 4. Discussion

In order to identify the trends of existing research related to sleep and eating disorders, a DCA network was generated. Seven major clusters were automatically identified. In this section, each cluster will be discussed in detail in ascending chronological order of average year of publication. Both citing articles and references cited will be included when analysing the clusters with the coverage (number of papers within the cluster referenced by the article) and global citing scores (GCS; i.e., the cumulative count of references received by an article within Scopus).

### 4.1. Cluster #9: Sleep-Related Eating Disorder

Cluster #9 includes papers that were published on average in 2005. The three major citing articles in this cluster were authored by Tzischinsky et al. [51] (coverage = 13; GCS = 4), Howell [52] (coverage = 12; GCS = 0), and Benca and Schenck [53] (coverage = 11; GCS = 12). Cluster #9 focuses on the fundamental concepts and research profile of sleep-related eating disorder and its related terms, as well as drug induction and pharmacological treatment. For instance, Tzischinsky et al. [51] discussed the background and concept of night eating syndrome and sleep-related eating disorder, as well as the relationship between night eating syndrome, eating disorders, and sleep-related eating disorder. Both sleep-related eating disorder and night eating syndrome are eating disorders that occur during the sleep period, and they are often found to be comorbid. In particular, sleep-related eating disorder is characterised by frequent occurrences of eating during the transition from night-time sleep to arousal. In sleep-related eating disorders, these eating episodes are associated with partial to very low levels of consciousness [54]. Night eating syndrome is characterised by instances of hyperphagia at full arousal from nocturnal sleep [54]. Although highly comorbid, sleep-related eating disorder and night eating syndrome are interpreted as the two poles in the disordered eating *continuum*. As argued by Howell et al. [45], night eating syndrome is derived from the combination of the atypical circadian rhythms of eating times with the typical circadian timing of sleep onset. The opposite pattern describes sleep-related eating disorder. In addition, many cited references focused on studying sleep-related eating disorder, particularly its pathogenesis, drug triggers, and medication treatment [43,44,49,55]. Vetrugno et al. [43] stated that a dysfunction in the dopaminergic system contributes to the pathogenesis of sleep-related eating disorder. Morgenthaler and Silber [44] also reported zolpidem’s effect in triggering sleep-related eating disorder among patients with potential sleep disorders. Winkelman [55] found topiramate effective in reducing nocturnal eating in patients with a chronic sleep-related eating disorder, while Provini et al. [49] reported Pramipexole’s effect in improving sleep quality and decreasing median night activity in sleep-related eating disorder. Furthermore, several cited articles were studies on night eating syndrome (e.g., [56,57,58,59]), which may be because night eating syndrome and sleep-related eating disorder are distinct yet related, as both involve eating at night, a chronic course, familial associations, comorbid neuropsychiatric disease, and are frequently related to weight gain and obesity [51].

### 4.2. Cluster #3: Nocturnal Eating

Cluster #3 consists of articles with a mean year of publication in 2007. The top three citing articles in this cluster were authored by Howell [60] (coverage = 24; GCS = 87), Howell and Schenck [61] (coverage = 16; GCS = 45), and Schenck and Mahowald [62] (coverage = 11; GCS = 2). Cluster #3 primarily focuses on nocturnal eating, which is a symptom associated with sleep-related eating disorder, night eating syndrome, and eating disorders [51]. In the cluster, some cited papers are related to studies on rapid or non-rapid eye movement sleep (e.g., [43,63,64,65]), possibly due to a relationship between nocturnal eating episodes and non-rapid eye movement sleep identified by some studies [43,66]. Other cited papers focus solely on nocturnal eating alone, with most concentrating on sleep-related eating disorder and night eating syndrome (e.g., [44,45,67,68,69]). In their suggestions for treatments of parasomnias in adults, Schenck and Mahowald [62] emphasised the importance of treating comorbid conditions and eliminating suspected inducing factors to address dysfunctional nocturnal eating. For instance, they suggested that dopaminergics, opioids, and benzodiazepines can effectively treat nocturnal eating associated with restless leg syndrome, a condition characterised by discomfort to the lower limbs of the body and by the urge to move them [61]. Many of the cited documents (e.g., [70,71,72,73,74]) focus on the association between nocturnal eating and restless leg syndrome. This is because nocturnal eating is interpreted as a non-motor manifestation of restless leg syndrome [60,61,62].

### 4.3. Cluster #46: Ghrelin

Cluster #46 is the smallest cluster in the network, containing only one citing paper authored by Wittekind and Kluge [75], with a coverage of 11 articles and a GCS of 66. Ghrelin, a peptide hormone produced in the stomach, is considered to be an orexigenic hormone that can stimulate hunger and appetite, as well as promote food intake. It is also considered to be involved in the regulation of physiological processes such as sleep, mood, memory, and reward. Wittekind and Kluge [75] highlighted the significant impact of ghrelin on the pathophysiology of addictive disorders, where it can enhance drug-seeking behaviour, promote drug reward, and increase craving in both animals and humans. A growing interest in the role played by ghrelin in eating and sleep disorders was also reported [76]. In patients with anorexia nervosa, as compared to healthy individuals, several studies have found increased levels of plasma and serum ghrelin (e.g., [77]). Interestingly, the increase in levels of ghrelin in anorexia was also found to be enhanced by cognitively restrained eating and by exposure to pictures of food [78,79]. Scholars interpret the increased levels of ghrelin in anorexia as a compensatory mechanism for chronic energy deficiency [75]. Differently, for bulimia nervosa, results about fasting ghrelin are contradictory, with some studies finding differences in ghrelin plasma levels in patients with bulimia nervosa as compared to healthy controls [80], and other studies not finding any difference between the two groups [75,81]. However, some studies seem to suggest that the abnormal food intake in bulimia nervosa might be triggered by a slow postprandial decline in ghrelin levels [82]. Finally, similar results were obtained for binge eating disorder, where patients seem to have constantly low basal serum levels of ghrelin as well as a slower decline in their ghrelin levels after a meal [75,83]. Few studies have also looked at the association between ghrelin and insomnia, with the results showing that patients with insomnia tend to have lower nocturnal levels of ghrelin as compared to matched controls [84].

### 4.4. Cluster #4: Comorbidities

The two major citing articles in cluster #4 were written by Kucukgoncu et al. [85] (coverage = 19; GCS = 27) and Allison et al. [2] (coverage = 15; GCS = 62). The main theme of this cluster pertains to the comorbid relationship between sleep disorders, eating disorders, and other emotional issues. For instance, Kucukgoncu et al. [85] showed that patients with night eating syndrome exhibit a higher risk of experiencing sleep disorders, depression, and anxiety. Similarly, Allison et al. [2] suggested a correlation between insomnia and an increased risk of eating disorders, as well as between eating disorders and disrupted sleep. The cited documents (e.g., [56,86,87,88,89]) corroborate these findings and confirm that the type and severity of comorbidities (including depression, anxiety disorders, mood disorders and sleep disorders) have a positive correlation with the severity of eating disorder symptoms. Additionally, some of the cited articles proposed other comorbid relationships such as night eating syndrome and restless leg syndrome [90].

### 4.5. Cluster #22: Mental Disorders in Sport

Cluster #22 includes three citing papers, authored by Gouttebarge et al. [91] (coverage = 12; GCS = 13), Gouttebarge and Kerkhoffs [92] (coverage = 11; GCS = 11), and Gouttebarge et al. [93](coverage = 8; GCS = 25). The cluster primarily focused on mental disorders among sports-related practitioners. In their works, a significant prevalence of symptoms of common mental disorders was revealed among professional football referees and elite athletes, particularly related to anxiety and depression [91,93]. The study on professional ice hockey players conducted by Gouttebarge and Kerkhoffs [92] identified a high prevalence of eating disorders among current players and of sleep disturbances among retired players. They also suggested that stressors such as a higher number of surgeries, a higher number of recent life events, and a higher level of career dissatisfaction were associated with symptoms of common mental disorders. Similarly, many of the cited documents (e.g., [94,95,96]) were studies that investigated the prevalence of common mental disorders among athletes in different countries and professions and arrived at similar conclusions as the citing papers above. Additionally, some of the cited papers focused on examining the organisational stressors that lead to common mental disorders among athletes, as well as the barriers and facilitators to help-seeking for young athletes in the general community [97,98].

### 4.6. Cluster #1: Sleep Quality in Anorexia Nervosa

The main citing articles in cluster #1 were written by Vinai et al. [99] (coverage = 11; GCS = 17), Nagata et al. [100] (coverage = 7; GCS = 6), and Ralph-Nearman et al. [101] (coverage = 7; GCS = 5). In the cluster, many references showed that patients with anorexia nervosa often tend to report sleep disturbances [2,100,102,103]. Interestingly, Abdou et al. [102] used subjective and objective (i.e., polysomnography) methods to measure sleep profiles in patients with anorexia and bulimia nervosa. The results of this study showed impairment in most domains of sleep measured by subjective and objective tools in patients with anorexia and bulimia nervosa as compared to healthy controls. Similarly to other types of eating disorders, patients with anorexia nervosa in some cases report episodes of night eating, which, in turn, are generally associated with poor sleep quality [99]. Across diagnostic groups in eating disorders, Vinai et al. [99] showed that people that report episodes of nocturnal eating have lower sleep efficiency and duration due to moderate sleep fragmentation measured using polysomnography. Using network analysis on data from 267 patients with anorexia nervosa, Ralph-Nearman et al. [101] showed that sleep- and anxiety-related symptoms are pivotal and strongly connected to the symptoms of anorexia nervosa. The authors also suggest that targeting sleep disturbances, anxiety, and worry might improve traditional treatments for anorexia nervosa. Some other cited papers in the cluster (e.g., [104,105,106,107,108]) are related to studies on night eating syndrome.

### 4.7. Cluster #17: Bidirectional Relationship

Cluster #17 includes three citing articles by Buelow [109] (coverage = 18; GCS = 11), Christensen and Short [110] (coverage = 14; GCS = 8), and Christensen et al. [111] (coverage = 13; GCS = 2). This cluster centres on research exploring insomnia in patients with eating disorders. Christensen and Short [110] described the bidirectional association between sleep and eating processes, and proposed a promising model for the underlying mechanisms. They suggested that acute symptoms of insomnia can worsen problematic eating behaviours, and eating disorder behaviours can alter sleep, leading to a positive feedback loop resulting in cognitive, physiological, and behavioural changes that can further entrench individuals in eating disorders and result in insomnia disorder. In agreement with the view that eating and sleep disorders influence one another in a loop cycle, Christensen et al. [111] proposed to evaluate an empirically supported treatment for sleep disturbances in patients with residual insomnia disorder after eating disorder treatment. Many of the cited documents in this cluster (e.g., [103,112,113,114,115,116]) focus on insomnia, including its comorbidities (e.g., panic disorder with agoraphobia and generalized anxiety disorder), treatment (e.g., cognitive behaviour treatment), as well as its predictive effect on mental disorders (e.g., depression, anxiety and psychosis). In particular, Lombardo et al. [103] showed that poor quality of sleep is a reliable predictor of the severity of eating disorders, with the mediation of depressive symptoms. Furthermore, the same pattern was observed after 6 months of standard treatment for eating disorders. Some cited papers explore the relationship between insomnia and eating disorders such as binge eating disorder (e.g., [114]). For instance, Kenny et al. [114] observed that insomnia symptoms are more frequent in patients with binge eating disorder as compared to people with no diagnosis of an eating disorder. Anxiety seems to mediate the association between insomnia and binge eating disorder, with depression working as a mediator between the severity of insomnia and binge frequency. Interestingly, a large number of the cited papers (e.g., [115,117,118,119]) also investigate substance abuse, as poor sleep quality caused by insomnia can increase the risk of substance addiction and relapse.

### 4.8. Limitations of the Study

The scientometric approach used in this study has some limitations. One of the main limitations is that, similarly to previously published scientometric reviews (e.g., Cortese et al. [120], Sabe et al. [121] and Zakaria and Aryadoust [122]), the sample of documents was collected from one platform, which in our case was Scopus. This means that some relevant documents that are published in journals not indexed in Scopus may have not been included in the current analysis. However, it is worth noting that merging references retrieved from different databases represents a non-trivial challenge for researchers given the differences between platforms. In the current state, merging references from different platforms is possible only with a significant degree of manual intervention, as no software allows this procedure to be conducted automatically [120]. Additionally, DCA only quantifies the number of citations and co-citations in the dataset and does not offer qualitative insights into the co-citation patterns. To address this limitation, this study also includes a qualitative discussion of the clusters following the scientometric analysis [42,123].

## 5. Conclusions

Our study employs a scientometric approach to review the extensive literature on sleep and eating disorders. Our study aimed to identify major thematic domains and significant publications and to discuss research gaps in this field using DCA. From the thematic clusters of research, a bidirectional relationship between eating disorders and sleep abnormalities strongly emerges. When modelling the reciprocal influence of eating and sleeping rhythms, the role played by pharmacological, hormonal, and psychological factors was elucidated. However, many of the observations on the reciprocal influence between sleep and eating patterns were obtained by means of subjective methods of sleep assessment, mainly based on self-report measures. Indeed, it seems that objective methods for evaluating sleep quality in individuals with eating disorders, such as polysomnography or actigraphy, are lacking. Some exceptions are the studies by Abdou et al. [102] and Vinai et al. [99], in which polysomnography was used in combination with self-subjective measures of sleep. The studies reviewed in this article outline the need to carry out objective sleep assessments in combination with hormonal assessments to find possible mechanisms of interaction between eating and sleep disorders. Elucidating these factors would allow for designing tailored lines of treatment for patients with eating disorders by taking into account both abnormal eating patterns and impaired sleep quality.

## Figures and Tables

**Table 1:** Summary metrics for the seven major clusters identified in the document co-citation analysis network.

Cluster ID	Size	Silhouette	Mean Year	LLR Label	Suggested Label
1	69	0.956	2014	sleep-related eating disorder	Sleep Quality in Anorexia Nervosa
3	57	0.970	2007	restless nocturnal eating	Nocturnal Eating
4	56	0.973	2010	depressed patient	Comorbidities
9	48	0.991	2005	sleep-related eating disorder	Sleep-Related Eating Disorder
17	34	1.000	2015	psychological disorder	Bidirectional Relationship
22	25	0.995	2013	common mental disorder	Mental Disorders in Sport
46	11	0.999	2010	psychiatric disorder	Ghrelin

**Table 2:** Nine documents with citation burstsness.

References	Citation Burstness	Publication Year	Burst Begin	Burst End	Duration	Centrality	Sigma
Vetrugno et al. [43]	8.3575	2006	2007	2012	5	0.00	1.03
Morgenthaler and Silber [44]	6.7376	2002	2005	2009	4	0.00	1.03
Howell et al. [45]	5.9064	2009	2010	2012	2	0.00	1.01
Winkelman et al. [46]	5.155	1999	2002	2006	4	0.00	1.00
Allison et al. [2]	4.7939	2016	2017	2021	4	0.01	1.04
American Psychiatric Association [47]	4.577	2013	2018	2021	3	1.01	1.04
Winkelman [48]	4.4768	2006	2009	2012	3	0.00	1.00
Provini et al. [49]	4.1406	2005	2011	2013	2	0.00	1.00
Winkelman [50]	3.9927	2003	2005	2011	6	0.00	1.00

## Data Availability

Not applicable.

## Supplementary Materials

The following supporting information can be downloaded at https://www.mdpi.com/article/10.3390/healthcare11142090/s1.

## Abbreviations

The following abbreviations are used in this manuscript:

DCA	Document Co-Citation Analysis
PRISMA	Preferred Reporting Items for Systematic Reviews and Meta-Analyses
LLR	Log-Likelihood Ratio
GCS	Global Citing Score
